# Editorial to the Special Issue “Plant Extracts: From Extract Technology to Health Benefits”

**DOI:** 10.3390/foods13030356

**Published:** 2024-01-23

**Authors:** Giuseppina Crescente, Stefania Moccia

**Affiliations:** National Research Council, Institute of Food Sciences, 83100 Avellino, Italy; giuseppina.crescente@isa.cnr.it

The valorization of food industry waste is essential to the sustainable development of the agro-food industry, starting from the extraction of plant special metabolites, a challenge that still exists today [1].

In this context, the Special Issue, entitled “Plant Extracts: From Extract Technology to Health Benefits”, contains four research papers and two reviews, covering the latest advances in the valorization of food by-products, from the extraction process to the validation of their functional activity in experimental models. 

Traditional and non-traditional extraction methods have been developed over the years. An ever-increasing number of extraction methods referred to as “green extraction” are currently being utilized in an effort to minimize the use of organic solvents and replace them with more eco- and environmentally friendly ones [2]. Green chemistry-based extraction techniques offer several advantages over conventional methods, including reduced extraction time, solvent consumption, etc., and fit with eco-extraction practices [3]. 

A novel technique, as Microwave Hydrodiffusion and Gravity (MHG) was applied by Crescente et al. (contribution 1) to recover high-value components from the Grape Pomace (GP) of a *Vitis vinifera* cultivar named “Aglianico”.

MHG is a quick, efficient, and environmentally friendly method of extraction without using solvents [4], compared to Ultrasound-Assisted Extraction (UAE) to recover polyphenol compounds from GP. A fractionation protocol was applied to obtain, for each parental extract, three purified fractions (F1, F2 and F3) which were characterized by spectroscopic techniques. As a result, MHG fractions showed increased radical scavenging performance when compared to UAE fractions, and MHG-enriched anthocyanin fractions (F3) decreased proliferation of HT-29 cells by 70%, suggesting the need to fully exploit their potential. Crescente et al. provide for the first time the biological validation of polyphenol fractions from GP obtained by MHG, which may serve as a good alternative to UAE for extracting polyphenols. Moreover, the significance of this study lies in the potential applications of this green technology to one of the most significant agro-food chains, the wine chain, which contributes significantly to global economic growth, through the recovery and valuation of waste materials from the production process. 

Water is typically used as a solvent in green extraction methods [5], usually in conjunction with a physical aid such as a microwave, rotatory water path, ultrasound, etc.

In this context, Alqahtani et al. (contribution 2) optimized a water bath-assisted extraction for two cultivars of date palm fruits (Anbara and Reziz). Through the application of response surface methodology, three “factors” (time, temperature, and rotation) were adjusted to achieve the maximum content of total phenols, total flavonoids, reducing power, and scavenging activity of the extract’s responses. As a result of the improvement of the extraction process, extracts with high phenolic and flavonoid content and antioxidant potency were produced. They highlighted that the two cultivars’ optimized date palm fruit extracts, inhibited CCl_4_-induced hepatic damage and had an anti-fibrotic impact. The results of this study can lead to the development of date palm fruit extracts enriched with phenolic and flavonoid compounds with interesting antioxidant properties, suggesting the potential application of date palm fruit extracts for liver disease treatment.

Water has been used also for the preparation of si-wu extracts (SWE) which turned out to be rich in (E)-ligustilide, chlorogenic acid, and verbascoside. Si-wu consists of four herbs, including *Angelica sinensis* (Oliv.) Diels, the processed root of *Rehmannia glutinosa Libosch.*, the rhizome of *Ligusticum chuanxiong* Hort., and the root of *Paeonia lactiflora f.* pilosella (Nakai) Kitag. SWE effectively improved mucus barrier damage in high-fat diet (HFD)-fed mice and mitigated colon diseases. HFD-fed mice supplemented with SWE for long periods decreased body weight and blood lipids, mitigated ER stress, improved O-glycosylation, and maintained mucin secretion. The results of this study provide a basis for further research for applying SWE in intestinal diseases related to mucosal damage (contribution 3). 

The neuroprotective effect of the aqueous extract of white *Nelumbo nucifera* Gaertn. petal tea was studied by Intui et al. (contribution 4). They have carefully examined the extract’s effect on cognitive behavior, hippocampus histology, oxidative stress, and amino acid metabolism in mancozed-poisoned rats, finding that the level of neuroprotective picolinic acid increased at the highest dose tested (2.20 mg/kg). On the other hand, the co-administration of *N*. *nucifera* extract with mancozed at a low dose (0.55 mg/kg) protected against mancozed toxicity suggesting its neuroprotective efficacy.

Anthocyanin belongs to the class of polyphenols and their ingestion has been correlated with a lower risk of cardiovascular disease [6]. Despite their health benefits, anthocyanins have a low bioavailability as opposed to their degradation products, referred to as phenolic metabolites [7,8]. To overcome this limitation, Festa et al. (contribution 5) have deeply investigated this topic in a review suggesting that microencapsulation can be a useful alternative to slowly release them into the gastrointestinal tract enhancing their health benefits in cardiovascular disease prevention and treatment. At concentrations that can be achieved in vivo, anthocyanin metabolites can improve vascular health. For this reason, more research on anthocyanin metabolites in synergistic action is recommended, including how they can be made more bioavailable and used as nutraceuticals. 

Encapsulation has lately gained popularity in the food sector [9]. The second review article enclosed in the present paper collection (contribution 6) focused its attention on the extraction and encapsulation processes of bioactive compounds from *Moringa oleifera* Lam. and their cytotoxicity. All parts of this plant are rich in bioactive molecules (carotenoids, phenolic compounds, alkaloids, glucosinolates, isothiocyanates, folates, tannins, saponins, and fatty acids) with multiple health-beneficial effects (anticancer, antibacterial, antiproliferative, antihypertensive, and anti-inflammatory) [10]. The authors concluded that by using green extraction and spray-drying as the most suitable methods to encapsulate bioactive compounds, *M. oleifera* could find broad application in various fields overcoming concerns about their cytotoxicity. 

To conclude, the contributions to this Special Issue outline some environmentally friendly methods for maximizing extraction procedures and recovering bioactive compounds from agro-food byproducts, emphasizing the need for further research in this area. This Special Issue includes valuable contributions from authors, which we hope readers will find informative and interesting.
